# Home treatment and use of informal market of pharmaceutical drugs for the management of paediatric malaria in Cotonou, Benin

**DOI:** 10.1186/s12936-018-2504-1

**Published:** 2018-10-10

**Authors:** Edwige Apetoh, Marina Tilly, Carine Baxerres, Jean-Yves Le Hesran

**Affiliations:** 1 Institut de recherche pour le développement, Unité mixte de recherche 216: Mères et enfants face aux infections tropicales, Université Paris-Descartes, 4 Avenue de l’Observatoire, 75006 Paris, France; 2 Ecole doctorale Pierre Louis de santé publique, ED 393 Epidémiologie et Sciences de l’Information Biomédicale, Paris, France; 3 Centre Norbert Elias EHESS-Campus Marseille La Vieille Charité, 2 Rue de la Charité, 13002 Marseille, France

**Keywords:** Benin, Cotonou, Home treatment, Informal market of pharmaceutical drugs, Malaria, Parallel market, Paediatric malaria, Self-medication

## Abstract

**Background:**

Malaria is the main cause of hospital admissions in Benin and a leading cause of death in childhood. Beside consultations, various studies have underlined the management of the disease through home treatment. The medicines used can be purchased in informal market of pharmaceutical drugs (IMPD) without prescription or any involvement of healthcare professional. Pharmaceutical drugs are sold by informal private vendors, who operate at any time in the immediate environment of the patients. The present study was conducted in Cotonou to study the health-seeking behaviour of caregivers to treat malaria in children under 12 years old. Factors associated with malaria home treatment and drugs purchase in IMPD were studied.

**Methods:**

A cross-sectional study was carried out among 340 children’s caregivers who were interviewed about their socio-demographic characteristics and their care-seeking behaviour during the most recent episode of malaria in their children under 12. Medicines used and purchase place were also collected. Multivariate logistic regression model was used to determine factors associated with malaria home treatment and drug purchase in IMPD.

**Results:**

Beyond all the 340 caregivers, 116 (34%) consulted healthcare professional, 224 (66%) home treat the children, among whom 207 (61%) gave pharmaceutical drugs and 17 (5%) gave traditional remedies to children. Malaria home treatment was associated with family size, health insurance (OR = 0.396, 95% CI 0.169–0.928), and wealth quintiles where home treatment was less used by the richest (OR = 0.199, 95% CI 0.0676–0.522) compared to those in the poorest quintile. The caregivers age group 30–39 years was associated to the use of IMPD (OR = 0.383, 95% CI 0.152–0.964), the most economically wealthy people were less likely to use IMPD (wealth quintile richest: OR = 0.239, 95% CI 0.064–0.887; wealth quintile fourth OR = 0.271, 95% CI 0.100–0.735) compared to those in the poorest quintile. All caregivers who benefited from health insurance did not use IMPD.

**Conclusion:**

This study highlights the link between worse economic conditions and accessibility to medical care as one of the main factors of malaria home treatment and drug purchase in IMPD, even if those two phenomena need to be understood apart.

**Electronic supplementary material:**

The online version of this article (10.1186/s12936-018-2504-1) contains supplementary material, which is available to authorized users.

## Background

Malaria is the leading cause of morbidity among adults, and mortality among children under five in Benin. It accounted for 40% of outpatient consultations and 25% of hospital admissions in 2016 [1]. In the context of the extended resistance to chloroquine and sulfadoxine–pyrimethamine (SP), the malaria management policy in Benin has been modified in 2004 by introducing into the national malaria coordination programme the use of artemisinin-based combination therapy (ACT) to treat non-complicated malaria [2]. This change was made 2 years before the official recommendation of the World Health Organization (WHO) to most sub-Saharan African countries [3]. In 2011, in Benin recommended malaria diagnosis [rapid diagnostic test (RDT) or microscopy] for all patients, as mandated by the diagnostic guidelines, and treatment being given only to patients with a positive test result [4].

Early diagnosis and prompt treatment hugely reduces progression of the illness to severe state and prevents death [5]. However, in Benin like in other sub-Saharan Africa countries, caregivers first manage malaria in children at home, and when the symptoms persist a health-care professional is consulted [6–9].

In Benin, malaria RDT is performed only after consulting health-care professional. Thus, home treatment of malaria in children is based on perceived symptoms by caregivers. It is well recognized that home treatment of malaria presents risk of false diagnosis and increased delay in presentation at health-care centres [6]. Home treatment was operationally defined as the administration of treatment (pharmaceutical drugs or traditional remedies) at home by the caregiver on his own initiative or on the advice of a relative, as opposed to treatment recommended by health-care professional on the present episode of disease [10]. Home treatment risks are increased in environments where people have access to a wide range of pharmaceutical drugs, even for drugs normally requiring prescription by a healthcare professional. Drugs can be bought in private or public formal sectors (pharmacies, health centres, private warehouses), but also in informal market of pharmaceutical drugs (IMPD). The selling of informal pharmaceutical drugs is a widespread practice since at least the 1980′s in several African countries, particularly in the French-speaking ones [11–13].

Informal refers to activities involving the sale and purchase of drugs outside the legislative and administrative framework imposed by a country’s government and biomedical health system. Pharmaceutical drugs are sold by informal private vendors, who operate as shop keepers, traders, itinerant drug sellers, and drug wholesalers [14–16]. This informal practice is taking place in a context marked in part by the lack of rigidity in executive, administrative or other measures, including penalties against sellers and distributors [17, 18]. In Benin, the procedures against IMPD have been mentioned in the national pharmaceutical policy [19]. On a related subject, a national counterfeit medicines committee was created in 2012 and actions, including awareness campaigns and repression through the seizure and destruction of drugs on the informal market were conducted. Since February 2017, an unprecedented repression was organized by the president of the Republic of Benin.

The last quantitative survey on the use of IMPD was conducted in Cotonou, the biggest town of Benin, in 2003, 15 years ago. The survey aimed to determine the percent of the population which at least once have purchased pharmaceutical drugs in IMPD. The percent reported was 72%, all diseases taken into account [20].

Cotonou is the city in which IMPD is mostly present due to the biggest West African international market named “Dantokpa”, where drugs are sold wholesale and retail [16]. In the order to describe malaria management in households in this environment, a household cross-sectional study was conducted. The study aimed to (i) describe the management of the most recent episode of malaria among children under twelve, (ii) document the pharmaceutical drugs used by caregivers to treat children, (iii) describe factors associated with malaria home treatment, and (iv) describe factors associated with the use of IMPD when home treatment was practiced.

## Methods

### Study area

This study was carried out before the 09th edition of Operation PANGEA IX in 2017, coordinated by Interpol with a view to curbing the informal circuit of pharmaceutical drugs in Benin. The study was conducted in Cotonou, the economic capital and biggest city of Benin. The country is divided in twelve departments and Cotonou forms the department of Littoral on its own. The general population of Cotonou was estimated at 679,012 in 2013, with 211,192 children under 12 years old [21]. Cotonou is a coastal city, limited to the west by the Atlantic department and to the east by Ouémé department. The Atlantic ocean is its southern boundary and the Nokoué lake is its northern boundary. This geographic situation makes malaria endemic in the Cotonou area. The primary malaria vector is *Anopheles gambiae* sensu stricto [4]. Severe malaria was the leading cause of death (24%) among children under 5 in hospitals in 2016 [22].

In Benin, the use of ACT to treat mild malaria was recommended by the health ministry of Benin since 2004 [2]. The National Malaria Control Programme of Benin recommended in 2011 new guidelines for effective malaria prevention and treatment [4]. Thus, patients of all ages should receive a confirmed malaria diagnosis [with either a rapid diagnostic test (RDT) or microscopy] before receiving treatment. Treatment is free of charge for pregnant women and children under 5 attending public health facilities, and the ACT subsidy system has been implemented in the public sector for other categories of patients. The subsidized ACT, artemether and lumefantrine, can be made available through different trade names, including Coartem^®^ or Lumartem^®^. Prevention strategies involve the use of insecticide-treated mosquito nets (ITNs), indoor residual spraying (IRS), and intermittent preventive treatment of pregnant women (IPTp).

### Study population

Caregivers were identified in households and asked about the treatment-seeking behaviour for the most recent malaria episode in a child under 12 years under their care. As self-medication is virtually non-existent in this age group, caregivers have full picture of pharmaceutical drugs consumed by children. This hypothesis was consolidated by a previous study. Geissler et al. [23] studied self-medication practice among schoolchildren aged 11–17 years in western Kenya, and reported that self-medication decreases with a younger age. Twenty percent of the 15–17 years old were practicing self-medication without parental or adult involvement. The prevalence was 14% among the 13–14 years old, and 9% among the 11–13 years old.

### Study design and sample size

A cross-sectional study was conducted in Cotonou, during April–May 2016. The numbers of caregivers to interview was calculated by the Schwartz’ formula [24]:

$$n = \frac{{\left( {z^{2} } \right)\left( {\hat{p}} \right)\left( {1 - \hat{p}} \right)}}{{e^{2} }},$$

where *z* represents the z-score fixed at 1.96; *e* indicates the desired margin of error fixed at 0.05 [25], and $\hat{p}$ the proportion of children under 12 which have already contracted malaria. This latter proportion was unknown, and was fixed to 50% in order to maximize the sample size [26]. A total of 384 caregivers of children under 12 should be included in the study.

Because of the absence of relevant data to perform a simple random sampling, a GPS sampling method was used to access caregivers. The sampling method consisted of random selection of a calculated number of GPS points into the study area. Buildings identified by GPS points were visited for the survey. Every GPS point may not necessarily identify a household because of empty spaces (park, field, swamps, or puddles) and uninhabited buildings (commercial, company or administrative buildings, buildings under construction or abandoned). Simulations performed determined that two-thirds of GPS points will identify a building. Considering that 1 out of 2 households would include at least one child less than 12 years of age, twice as many households should be visited. The number of GPS points to randomize to reach 384 caregivers of children under 12 years old was calculated with the formula:

$$n_{GPS} = n \times 1/p_{child} \times 1/p_{building} \times 1/p_{{non-response}} ,$$

where *n* was the calculated number of children to include in the study, *p*_*child*_ the proportion (1/2) of households with at least one child under 12, *p*_*building*_ the proportion (2/3) of GPS points which identifies a building, and *p*_*non*-*response*_ the assumed proportion (1/10) of non-response [26] *n*_*GPS*_ was 1300.

A total of 421 households contained at least a child under 12 and caregivers were interviewed about the most recent episode of malaria in a child under their care. A total of 340 (81%) caregivers were able to provide information on the health-care seeking behaviour and were included in the study. Forty-nine (12%) declared that the child has never contracted malaria, 23 (5%) declared that they practised prevention, so that children had not contracted malaria in the recent past, and 9 (2%) caregivers declared they did not remember.

### Collected data

Questionnaire for data collection was translated into the local dialect “fon”, the most common spoken in Cotonou by non-French speakers. In the households, caregivers were interviewed. A questionnaire was administered by investigators who graduated in sociology with substantial knowledge of the field surveys.

Questions were about the health-seeking behaviour during the most recent malaria episode among their children under 12 in the household. The episode date was collected and caregivers were asked to cite any symptoms which leaded them to recognize that the child had malaria. Afterward, the first action done by caregivers after they recognized symptoms were collected. Action could then be either home treatment or consultation with a healthcare professional. Information were collected on the type of treatments used at home or prescribed (pharmaceutical drugs or traditional remedies), treatment name and galenic form of pharmaceutical drugs. When home treatment was practised, the purchase place of pharmaceutical drugs was collected. Purchase places were categorized into IMPD and formal market.

A first binary outcome variable was home treatment of malaria, defined in this paper as treatment (pharmaceutical drugs or traditional remedies) administration at home by the caregiver on his own initiative or on the advice of a relative, as opposed to treatment recommended by health-care professional. For this purpose, used leftover medicines having been advised by pharmaceutical drugs vendors (pharmacist or informal vendor) were considered as home treatment.

A second binary outcome variable was drug purchase in IMPD or in formal market when home treatment was practised. Independent variables were, age, education level, marital status, religion, health insurance, family size, and presence of relatives living in the same inner court. A set of 17 variables on the household possession and housing characteristics (nature of habitat, access to resources and property owned by the household) were collected and facilitated the construction of a wealth index by using principal component analyses, like in the demographic and health survey (DHS) [27]. The wealth index was categorized into quintiles in ascending order of poorest, fourth, middle, second, and richest. However, the variables used in this study were not exactly similar to those of the demographic and health survey, and, therefore, the wealth quintile used in this study cannot strictly be compared to DHS data.

### Data analysis

Data were analysed using STATA version 13 statistical software (Stata Corp., TX USA). Descriptive analyses were performed to characterize socio-demographic aspects of caregivers, and management of the disease. Median and length were calculated for continuous variables and percentages for categorical variables. To assess the significance of differences between proportions the Chi square test or Fisher’s exact test, were used as appropriate.

Forgetting the name of drugs used over time can be a consequence of a memory time-related bias. To identify any possible memory time-related bias, the date of the most recent episode was quantified and grouped by months, and Chi square tests were conducted based on information related to the forgetfulness of the used treatments names, and secondly with therapeutic classes of cited drugs.

Bivariate logistic regression analyses were performed on the one hand with home treatment as the outcome, and in the other hand with drug purchase in IMPD as the outcome. For each outcome, all significant variables in the bivariate analyses at p < 0.20 were included in a multivariate logistic regression model. Odds ratios (OR) were reported with 95% confidence intervals (CI). Statistical significance was set at p < 0.05.

### Ethical considerations

The study has been approved by the Ministry of Higher Education and Scientific Research of Benin; Ethics Committee CER-ISBA –FAVORABLE ADVICE N° 30. Each interviewed person was informed about the objectives of the study, the types of collected data and were asked to sign a document of informed consent.

## Results

Among the 340 caregivers, 27% reported that children had malaria during the month preceding the interview, 36% had malaria in the last 1–3 months, 15% within the last 4–6 months, and 22% declared that the most recent episode dates back more than 6 months. The median time was 2 months. There was no evidence for memory time-related bias; neither for forgetfulness of the used treatment names, nor for the medicines therapeutic class (see Additional file 1). Based on these results, all data were included in subsequent analyses.

### Socio-demographic characteristics of caregivers

Table 1 presents the socio-demographic characteristics of caregivers and data on the management of the disease. Of the 340 interviewed caregivers, 75% were female and 80% were in a marital or non-marital relationship. Median age was 38 (range 18–83 years), 23% reported having received no formal education, 53% had primary education, 38% had attended secondary education and 9% had tertiary level education. Most caregivers were Christian (72%). Regarding family size, 28% of caregivers lived in households composed of at least three persons (four persons was the average size of households in Cotonou, according to the last census [21]), and 57% lived in households composed of 4–8 persons and 15% up to 8 persons. Thirty-one percent of the families lived in an inner court together with relatives. Thirty-eight percent of caregivers belonged to the upper third quintile, which characterizes the most economically wealthy people. Only 9% of caregivers reported that they subscribed to a health insurance. Seventy-nine percent of caregivers who brought their children to a healthcare professional reported that RDT or microscopy was done to confirm malaria.

**Table 1:** Socio-demographic characteristics of caregivers of under twelve children in Cotonou, Benin, May 2016 (N = 340)

Categories	Subcategories	Frequencies (n)	Percentages (%)
Age group (years)	< 30	63	18
	30–39	128	38
	40–49	68	20
	≥ 50	81	24
Gender	Female	255	75
	Male	85	25
Marital status	In relationship	273	80
	Single	67	20
Education	None	79	23
	Primary	100	53
	Secondary	130	38
	Tertiary	31	9
Religion	Christian	246	72
	Islam	47	14
	Other	47	14
Family size	< 4	38	28
	4–8	252	57
	≥ 9	50	15
Relative in the inner court	No	233	69
	Yes	107	31
Health insurance	No	310	81
	Yes	30	9
Wealth quintiles	Quintile 1 (richest)	57	17
	Quintile 2 (fourth)	70	21
	Quintile 3 (middle)	68	20
	Quintile 4 (second)	75	22
	Quintile 5 (poorest)	70	21

### Treatment-seeking behaviour and illness history

Table 2 presents the malaria health-seeking behaviours of caregivers and the symptoms in the child they recognized as malaria symptoms. To treat malaria, 116 (34%) caregivers indicated they brought the child to a healthcare professional, whereas 224 (66%) home-treated children. In the case of home-treatment, 203 (90%) reported they gave only pharmaceutical drugs to their child, while 17 (8%) reported they gave only herbal remedies. Only 4 (2%) caregivers declared giving both at the same time. As these later caregivers gave pharmaceutical drugs to the child, they were analysed together with those who used pharmaceutical drugs.

**Table 2:** Health-seeking behaviours of caregivers to treat malaria in children under twelve and malaria symptoms recognized in Cotonou, Benin, May 2016 (N = 340)

Categories	Subcategories	Frequencies (n)	Percentages (%)
Treatment-seeking	Healthcare professional	116	34
	Home treatment with pharmaceutical drugs	207	61
	Home treatment with traditional remedies	17	5
Fever, headache, dizziness	No	30	8
	Yes	310	91
Body aches, chills, weak	No	248	73
	Yes	92	27
Bitter taste in the mouth, no appetite	No	268	79
	Yes	72	21
Vomiting, diarrhoea	No	265	78
	Yes	75	22
Dark urine, yellow eyes	No	312	92
	Yes	28	8
Cough	No	310	91
	Yes	30	9
Other: prickly, sores in the mouth, anaemia	No	328	96
	Yes	12	4
Number of symptoms (md[length] = 2 [1,6])	1	141	42
	2	134	39
	≥ 3	65	19
Use of IMPD for home treatment with pharmaceutical drugs^a^ (n = 207)	No	123	59
	Yes	84	41

^a^Based on the number (n = 207) of caregivers who practised home treatment with pharmaceutical drugs

When asked about the symptoms that made them think that the child has malaria, at least five percent of caregivers reported fever (89%), loss of appetite (20%), vomiting (19%), weakness (17%), chills (10%), headache (9%), coughing (10%), and dark urine (6%). Forty-two percent of the caregivers cited only one symptom (30% fever), indicating they relied on a single symptom to conclude the child had malaria, 39% relied on two symptoms, and 19% on more than two symptoms. For the remainder of the analyses, cited symptoms were grouped into seven categories. The first category was “fever, headache, dizziness”, the second includes “body aches, chills, weak”, the third “bitter taste in the mouth, no appetite”, the fourth “vomiting, diarrhoea”, the fifth “dark urine, yellow eyes”, the sixth “cough”, and the last category regroups other symptoms which are rarely mentioned (“prickly, sores in the mouth, anaemia”).

### Factor associated with the malaria home treatment

Table 3 presents factors associated with malaria home treatment in bivariate and multivariate analyses. In bivariate analyses, caregivers who practised home treatment were significantly different from those who did not, in terms of age, education, family size, possession of health insurance, wealth quintiles and the fact that the identified symptoms were either “fever, headache, dizziness” and/or “vomiting, diarrhoea”.

**Table 3:** Factors associated with the practice of home treatment, in Cotonou, Benin, May 2016 (N = 340)

Covariates		Home treatment	Total	Bivariate			Multivariate		
Categories	Subcategories	n (%)	n	OR	95% IC	p-value	OR	95% IC	p-value
Age	< 30	40 (18)	63	1			1		
	30–39	87 (39)	128	1.220	0.647–2.298	0.538	1.382	0.678–2.815	0.372
	40–49	45 (20)	68	1.125	0.548–2.306	0.748	1.057	0.479–2.328	0.890
	≥ 50	52 (23)	81	1.031	0.519–2.045	0.930	0.959	0.447–2.054	0.915
Gender	Female	168 (75)	255	1					
	Male	56 (25)	85	1	0.595–1.678	1.000			
Marital status	Single	41 (18)	67	1					
	In relationship	183 (82)	273	1.354	0.782–2.341	0.278			
Education	None	58 (26)	79	1			1		
	Primary	68 (30)	100	0.769	0.400–1.477	0.431	0.699	0.236–2.069	0.518
	Secondary	79 (35)	130	0.560	0.304–1.033	0.064	0.627	0.230–1.709	0.362
	Tertiary	19 (9)	31	0.573	0.238–1.379	0.214	0.535	0.206–1.385	0.198
Religion	Christian	164 (73)	246	1					
	Islam	27 (12)	47	0.675	0.357–1.275	0.226			
	Other	33 (15)	47	1.178	0.597–2.324	0.635			
Family size	< 4	25 (11)	38	0.542	0.210–1.398	0.205	0.396	0.139–1.125	0.082
	4–8	160 (72)	252	0.490	0.239–1.004	0.051	0.399	0.180–0.885	*0.024*
	9–22	39 (17)	50	1					
Relative in the inner court	No	154 (69)	233	1					
	Yes	70 (31)	107	0.970	0.599–1.571	0.903			
Health insurance	No	211 (94)	310	1			1		
	Yes	13 (6)	30	0.358	1.677–0.767	0.008	0.396	0.169–0.9284	*0.033*
Wealth quintiles	Quintile 5 (poorest)	57 (25)	70	1			1		
	Quintile 4 (second)	50 (22)	75	0.456	0.211–0.985	0.046	0.470	0.207–1.065	0.071
	Quintile 3 (middle)	46 (21)	68	0.476	0.211–0.985	0.066	0.428	0.184–0.994	*0.049*
	Quintile 2 (fourth)	44 (20)	70	0.385	0.178–0.836	0.016	0.472	0.198–1.121	0.089
	Quintile 1 (richest)	27 (12)	57	0.205	0.092–0.454	0.000	0.199	0.076–0.522	*0.001*
Fever, headache, dizziness	No	23 (10)	30	1			1		
	Yes	201 (90)	310	0.561	0.233–1.349	0.197	0.477	0.176–1.286	0.144
Body aches, chills, weak	No	163 (73)	248	1					
	Yes	61 (27)	92	1.026	0.618–1.701	0.920			
Bitter taste in the mouth, no appetite	No	182 (81)	268	1					
	Yes	42 (19)	72	0.725	0.421–1.246	0.245			
Vomiting, diarrhoea	No	187 (83)	265	1			1		
	Yes	37 (17)	75	0.406	0.240–0.685	0.001	0.374	0.196–0.712	*0.003*
Dark urine, yellow eyes	No	205 (92)	312	1					
	Yes	19 (8)	28	1.101	0.482–2.519	0.818			
Cough	No	207 (92)	310	1					
	Yes	17 (8)	30	0.650	0.304–1.391	0.268			
Other (prickly, sores in the mouth, anaemia)	No	217 (89)	328	1					
	Yes	7 (3)	12	0.716	0.222–2.307	0.576			
Number of symptoms				0.691	0.534–0.894	0.005	0.864	0.629–1.186	0.368

Statistically significant p values are in italic

In multivariate analysis, caregivers who lived in a family composed of more than eight persons were more likely to home treat malaria in children, but the difference was only statistically significant when compared to family size in the 4–8 group (OR = 0.399, 95% CI 0.180–0.885). When caregivers had health insurance, they were less likely to home treat children (OR = 0.396, 95% CI 0.169–0.928) compared to those who did not have it. Caregivers belonging to the richest wealth quintiles were less likely to treat children at home (OR = 0.199, 95% CI 0.0676–0.522) compared to those in the poorest quintile. Home treatment were less practised by those who reported “vomiting, diarrhoea” (OR = 0.374, 95% CI 0.196–0.712) than those who reported other symptoms.

### Factors associated with the medicines purchased in IMPD

Table 4 presents factors associated with the use of IMPD in bivariate and multivariate analyses. It is important to underline that any caregiver who declared to have health insurance did not buy drugs in IMPD in this study.

**Table 4:** Factors associated with drug purchase in IMPD for home treatment (N = 207) in Cotonou, Benin, May 2016

Covariates		IMPD	Total	Bivariate			Multivariate		
Categories	Subcategories	n (%)	n	OR	95% IC	p-value	OR	95% IC	p-value
Age	< 30	21 (25)	36	1			1		
	30–39	27 (33)	80	0.363	0.162–0.816	0.014	0.383	0.152–0.964	*0.042*
	40–49	18 (21)	44	0.494	0.202–1.209	0.123	0.517	0.190–1.1403	0.195
	≥ 50	18 (21)	47	0.443	0.182–1.075	0.072	0.470	0.170–1.298	0.145
Gender	Female	60 (71)	154	1					
	Male	24 (29)	53	1.296	0.690–2.435	0.419			
Marital status	Single	15 (18)	37	1					
	In relationship	69 (82)	170	1.001	0.485–2.067	0.996			
Education	None	29 (35)	52	1			1		
	Primary	26 (31)	61	0.589	0.279–1.246	0.165	0.726	0.305–1.729	0.470
	Secondary	25 (30)	75	0.396	0.191–0.821	0.013	0.563	0.242–1.312	0.184
	Tertiary	4 (5)	19	0.211	0.061–0.724	0.013	0.419	0.094–1.857	0.253
Religion	Christian	59 (70)	153	1					
	Islam	11 (13)	26	1.168	0.502–2.715	0.718			
	Other	14 (17)	28	1.593	0.709–3.578	0.259			
Family size	< 4	6 (26)	21	1					
	4–8	64 (76)	150	1.860	0.684–5.059	0.224			
	> 8	14 (17)	36	1.590	0.498–5.074	0.433			
Relative in the inner court	No	56 (67)	143	1					
	Yes	28 (33)	64	1.208	0.665–2.195				
Wealth quintiles	Quintile 5 (poorest)	30 (36)	48	1			1		
	Quintile 4 (second)	23 (27)	45	0.142	0.274–1.433	0.269	0.860	0.350–2.108	0.742
	Quintile 3 (middle)	15 (18)	44	0.200	0.132–0.729	0.007	0.500	0.198–1.261	0.142
	Quintile 2 (fourth)	11 (13)	44	0.310	0.814–0.491	0.000	0.271	0.100–0.735	*0.010*
	Quintile 1 (richest)	5 (6)	26	0.627	0.045–0.445	0.001	0.239	0.064–0.887	*0.032*
Fever, headache, dizziness	No	2 (2)	18	1			1		
	Yes	82 (98)	189	6.130	1.370–27.417	0.018	9.384	1.807–48.730	*0.008*
Body aches, chills, weak	No	62 (74)	151	1					
	Yes	22 (26)	56	0.928	0.496–1.738	0.817			
Bitter taste in the mouth, no appetite	No	65 (77)	168	1					
	Yes	19 (23)	39	1.505	0.747–3.032	0.252			
Vomiting, diarrhoea	No	67 (80)	176	1			1		
	Yes	17 (20)	31	1.975	0.914–4.266		2.606	0.928–7.315	0.069
Dark urine, yellow eyes	No	79 (94)	191	1					
	Yes	5 (6)	16	0.644	0.215–1.927	0.432			
Cough	No	78 (93)	191	1					
	Yes	6 (7)	16	0.869	0.303–2.490				
Other: prickly, sores in the mouth, anaemia	No	82 (98)	202	1					
	Yes	2 (2)	5	0.975	0.159–5.967	0.979			
Number of symptoms				1.419	0.968–2.078	0.072	1.577	0.725–1.846	0.539

Statistically significant p values are in italic

*IMPD* Informal market of pharmaceutical drugs

In bivariate analysis, caregivers who purchased medicines in IMPD significantly differed from those who went to formal market in terms of age, education, wealth quintiles, and when reporting “fever, headache, dizziness”, “vomiting, diarrhoea”.

In multivariate analyses, caregivers belonging to age groups 30–39 (OR = 0.383, 95% CI 0.152–0.964) and economically wealthy people (wealth quintile richest: OR = 0.239, 95% CI 0.064–0.887; wealth quintile fourth OR = 0.271, 95% CI 0.100–0.735) were less likely to purchase drug in IMPD compared to those in the poorest quintile. When reporting “fever, headache, dizziness”, caregivers were more likely to go to IMPD.

### Types of drugs cited

Sixty-seven (20%) caregivers reported they forgot the name of the drugs used, 45 (38%) of whom consulted a healthcare professional, and 22 (11%) administered home treatment. Table 5 presents medicines reported by the 256 caregivers when a healthcare professional was consulted versus home treatment, and Table 6 presents location of treatment purchase in the case of home treatment.

**Table 5:** Pharmaceutical drugs used by caregivers to treat malaria in children under 12 in Cotonou, Benin, May 2016 (N = 256): home treatment versus healthcare professional

Variates		Healthcare professional	Home treatment	Chi square test
Categories	Subcategories	n = 71 (%)	n = 185 (%)	p-value
Therapeutic classes	Anti-malarial	63 (89)	120 (65)	*0.000*
	Antipyretic	30 (42)	125 (68)	*0.000*
	Anthelmintic	4 (6)	11 (6)	0.924
	Antibiotic	18 (25)	24 (13)	*0.017*
	Supplements	13 (18)	26 (14)	0.828
Anti-malarial	ACT	21 (30)	55 (30)	0.980
	SP	2 (3)	7 (4)	0.707
	Quinine	42 (59)	45 (24)	*0.000*
	Chloroquine	0 (0)	11 (6)	*0.036*
	Other anti-malarial	1 (1)	4 (2)	0.696
Galenic form	Tablet/pill	28 (39)	167 (90)	*0.000*
	Injection/infusion	39 (55)	1 (1)	*0.000*
	Syrup	25 (35)	29 (16)	0.019
	Other	7 (10)	7 (4)	0.157
Number of different therapeutic classes	1	22 (31)	84 (45)	*0.036*
	2	25 (35)	77 (42)	0.348
	≥ 3	24 (34)	24 (13)	*0.000*

Statistically significant p values are in italic

*ACT* artemisinin-based combination therapy, *SP* sulfadoxine–pyrimethamine

**Table 6:** Purchase place of drugs used by caregivers who practised home treatment to treat malaria in children under 12 (N = 185)

Variates		Formal Market	IMPD	Chi square
Categories	Subcategories	n = 118 (%)	n = 67 (%)	p-value
Therapeutic classes	Anti-malarial	81 (69)	39 (58)	0.153
	Antipyretic	71 (60)	54 (81)	*0.004*
	Anthelmintic	4 (3)	7 (10)	0.101
	Antibiotic	13 (11)	11 (16)	0.293
	Supplements	10 (8)	16 (24)	*0.004*
Anti-malarial	ACT	44 (37)	11 (16)	*0.003*
	SP	6 (5)	1 (1)	0.425
	Quinine	24 (20)	21 (31)	0.094
	Chloroquine	3 (3)	8 (12)	*0.019*
	Other anti-malarial	4 (3)	0 (0)	0.298
Galenic form	Tablet/pill	101 (76)	66 (93)	*0.003*
	Injection/infusion	1 (1)	0 (0)	1
	Syrup	25 (19)	4 (6)	*0.010*
	Other	6 (5)	1 (1)	0.425
Number of different therapeutic classes	1	60 (51)	24 (36)	*0.048*
	2	46 (39)	31 (46)	0.334
	≥ 3	12 (10)	12 (18)	0.132

Statistically significant p values are in italic

*IMPD* Informal market of pharmaceutical drugs, *ACT* artemisinin-based combination therapy, *SP* sulfadoxine–pyrimethamine

Different therapeutic classes were cited. Seventy-one percent of caregivers cited anti-malarial drugs, 61% antipyretics (analgesics or anti-inflammatory drugs), 16% antibiotics, 13% supplements (vitamins or anti-anaemics), and 6% anthelmintic drugs. Other therapeutic classes, including decongestants, cough or cold drugs, antidiarrhoeal drugs and antihistamines were also mentioned, but rarely. A total of 92 different trade names of pharmaceutical drugs were cited. The most cited drugs (by at least 5% of caregivers) were Paracetamol (44%), Quinine (32%), Efferalgan^®^ 10%, Amoxicillin (9%) and Lufanter^®^ (6%), which is the most cited ACT. Coartem^®^ (4 home treatments and 2 prescriptions) and Lumartem (2 home treatments and 1 prescription) were rarely mentioned.

### Home treatment *versus* prescription by a healthcare professional

Caregivers who brought their child to a healthcare professional more frequently reported the use of anti-malarial drugs (89%), compared to those who home treat children (65%) (p < 0.001). According to the type of the anti-malarial drug used, ACT was used in the same proportion in the case of home treatment and prescription (30%), while quinine was more often prescribed (p < 0.001) and chloroquine was more often used for home treatment (< 0.001).

A higher percentage of caregivers who treated children at home gave antipyretics (68%) than those who brought their child to a healthcare professional (42%) (p < 0.001). Antibiotics were more often cited when healthcare professionals were consulted (25%) than when home treatment was used (13%) (p < 0.05).

The most frequently therapeutic class used was anti-malarials when a healthcare professional had been seen (p < 0.001), and antipyretics in case of home treatment (p < 0.001). Concerning therapeutic classes used together, the pair anti-malarials-antipyretics was the most frequently used in home treatment, whereas anti-malarials-antibiotics was the most frequently prescribed. Concerning the galenic form, parenteral administration (p < 0.001) and syrup (p < 0.05) were used more in prescriptions, and tablet (p < 0.001) was preferred in the case home treatment.

### IMPD versus formal market

The places of purchase were recorded only in the case of home treatment, and not after consulting a healthcare professional. Table 5 presents data on locations provided by 185 caregivers who bought a pharmaceutical treatment. Various commercial drug names were cited, underlining the availability of a wide variety of pharmaceutical drugs available for purchase without prescription (see Additional file 2 for the name of pharmaceutical drugs cited).

There was no difference in the use of anti-malarials in relation to the purchase place. However, ACT (p < 0.05) was more often purchased in formal market, whereas chloroquine (p < 0.02) was more often purchased in IMPD. When drugs were used alone, anti-malarials (p < 0.001) was the most often purchased in the formal market. Caregivers who went to IMPD, were more likely to purchase more than two different therapeutic classes of drugs (p < 0.01). Concerning the galenic form, tablets (p < 0.01) were purchased more frequently in IMPD, and syrup (p < 0.05) in the formal market.

## Discussion

While home treatment is common it carries risks, whether individuals use medicines inappropriately, or whether its practice increases the delay in consulting a healthcare professional to receive appropriate care [28, 29]. Prompt diagnosis and treatment is the most effective means of preventing a mild case of malaria from developing into severe disease with increased risk of death [30]. In sub-Saharan African countries, home treatment has been widely documented as a common practice to treat illnesses, including malaria [13, 16, 31, 32]. Aside from the delay and the inappropriate use of medicines, home treatment presents risk in environment in which people can purchase medicines that are not subject to any government control (such as in the IMPD). This study sought to characterize the practice of malaria home treatment in children under 12 years old, and the use of the IMPD among caregivers who home treat malaria.

### Malaria home treatment

In this study, the prevalence of malaria home treatment (66%) is fairly close to the one previously reported (69.7%) by Agueh et al. [31] who considered it across all ages in Comé, a semi-rural area, 80 km far from Cotonou. This similarity is surprising. It would have been expected that home treatment was less frequent when treating malaria in children, knowing that on the one hand malaria treatment is free of charge for children under the age of five, and on the other hand the living environment were different in the two areas in terms of the health-care structures availability. However, as malaria is endemic in Benin, the similar prevalence can result from the fact that symptoms associated with malaria are very common and that people believe to know how to treat them. Consequently home-treatment is first used before any other action, including consulting health-care professional [33]. However, when considering the factors associated with malaria home treatment in this study (i.e. more than 8 members in the family, not having health insurance, belonging in the poorest quintile), the problem seems to rather be the lack of financial means prevailing in both rural and urban areas. Indeed, Agueh et al. also reported that the poorest quintile was associated with the highest home treatment rate. These results confirm those reported by several other studies showing that economic hardship was one of the main reasons cited by persons who home treated, both for malaria or other diseases [20, 34].

Following the Bamako Initiative, Benin, like other African countries, introduced out-of-pocket payments for health [35]. Healthcare that was hitherto free, became payable by the population. In Benin, it has been reported that 75% to 80% of households directly pay medical fees, and 76% of health spending is spent on pharmaceuticals and other medical goods [36]. This can cause households to incur catastrophic expenditures, which in turn can push them into poverty [37]. As this study shows that only very few caregivers declared to possess health insurance, obviously the financial expenditures increase with the number of persons in a family without health insurance, and could explain why the size of the household is associated with the practice of home treatment.

The proportion of caregivers who declared to have health insurance was very low (9%), even though health insurances have been operating in Benin since the 1990s [38], mainly for salaried employees, officials and army personnel, but not for informal workers. Unfortunately, health insurance’s contribution in relieving the financial burden on households remains low [38]. The majority covers only small risks (primary curative consultation, pre/post-natal consultation, normal childbirth, essential drugs) that are supported at the level of dispensaries, district health centres and communal health centres. In 2008, the health ministry launched the process of setting up a Universal Health Insurance Plan (Régime d’Assurance Maladie Universelle, RAMU), which would offer effective protection against the risk of illness to every Beninese whatever his social condition [35]. However, this project did not succeed, and was replaced in 2016 by the Insurance for Strengthening Human Capital [Assurance pour le Renforcement du Capital Humain (ARCH)] which will have to identify the essential dimensions of the Universal Health Coverage recommended by the WHO [36]. The fact that people who got health insurance practised less home treatment than those who got none, should encourage the government of Benin in the effective implementation of the ARCH.

Caregivers who declared that symptoms exhibited by the child were “vomiting, diarrhoea” practised home treatment less frequently. Diarrhoea was poorly known as a malaria symptom and was rarely mentioned in Tanzania [39] and Nigeria [40]. Furthermore, the proportions of respondents who perceived vomiting as a symptom of malaria varies across studies, from low (8.8%) in Lagos, Nigeria among pregnant women [41], to varying from 17.6% to 32.4% in households in four selected districts of the Central African Republic [42]. The fact that, caregivers who declared “vomiting, diarrhoea” practised home treatment less frequently might suggested that these symptoms were not always considered as symptoms malaria symptoms in also this study, thus necessitate referral to a health-care professional for a differential diagnosis.

Caregivers who home treat children more often remembered the name of medicines they used. This raises the question whether caregivers were being careful with prescriptions made by a healthcare professional. A study conducted on the Kenya coast reported that, among mothers who bring children to a healthcare professional, about 55% of them did not understand their prescription, yet none asked for clarification as they believed that health workers had no time for such explanations, and could become harsh when asked too many questions [43].

### Informal market of pharmaceutical drugs

The prevalence of the use of IMPD when home treatment was practised (41%) is lower in this study than in the study of Come (83.7%). This can be explained by environmental and economic factors. Results show that economic factors are associated with IMPD use. People from rural areas are generally poorer than those from urban areas. In the department Mono, where Come is located, the indicator of human poverty is almost twice that of Cotonou [21]. Additionally, the density of pharmacies is much higher in Cotonou (2017: 97 pharmacies over a surface of 79 km^2^) than in the Mono department (7 over a surface area of 1605 km^2^) [21, 22].

Without considering events that occurred after this study, the Ministry of Health of Benin conducted awareness campaigns in 2007 to curb IMPD in Benin, and since 2012, a strong appeal is made annually to inform populations about risks associated with consuming IMPD’s drugs. Yet, after these measures, the prevalence of IMPD use still seems high. More than one in three caregivers buy drugs in IMPD for home treatment of children under twelve.

The poorest people were the most likely to purchase drugs in the IMPD, as reported in Nigeria [44] and previously in Cotonou [20]. People without a fixed monthly income more frequently bought drugs in the IMPD, while a lower proportion of people with a higher income (more than 100,000 FCFA) buy IMPD drugs. However, some caregivers of the richest quintile purchased drug in IMPD, underlining that it is not exclusively a financial problem. Furthermore, the lack of difference in IMPD usage by caregivers belonging to the middle quintile and the poorest quintile adds complexity. It should also be noted that informal vendors of pharmaceutical drugs are usually part of the neighbourhood. As shown by Baxerres [16], each inhabitant of Cotonou potentially has an informal vendor of pharmaceutical drugs in his social or family circle (a friend of parents, a sister, a mother-in-law, a sister-in-law). The routine and trusting bonds which are being established between them could be the reason.

### Pharmaceutical drugs used

Various therapeutic classes of drugs were administered to children. Antipyretics were the most frequently used therapeutic class for home treatment in this study. This may not be surprising, considering that fever is the main symptom associated with malaria. However, fever can be also associated with virus infections that can be treated successfully with antipyretics. Therefore, some caregivers choose to use antipyretic drugs and not anti-malarial drugs to treat children. Fever can transiently decrease after treatment, but home treatment increases the delay of seeking appropriate health care, and may increase the risk of disease progression into severe malaria.

In this study, ACT was used in the same proportions both in the case of home treatment and by prescription. However, the first anti-malarial drug used for home treatment was a artemisinin-based combination, while quinine was the first prescribed drug. Parenteral administration was most frequently mentioned for prescriptions, supposing that the disease seemed severe and considering that caregivers who consulted a healthcare professional more often cited “vomiting, diarrhoea” as symptoms. The fact that health professionals prescribed antibiotics and anti-malarials together could highlight a lack of diagnosis (infection or malaria). Chloroquine was not prescribed, in compliance with recommendations, but continues to be used for home treatment in Cotonou. Use of chloroquine was also reported in Comé and other countries like Ghana or Nigeria [13, 45], where drug resistance has been confirmed, and where the use of ACT was implemented as a first-line drug to treat uncomplicated malaria.

### Synthesis

Children home treatment is not country specific. One important motivation for home treatment is the need for quick relief. Moreover, home treatment is prevalent when the illness is deemed to not require medical consultation or when it is considered as a familiar problem for which the drug is already known [6, 28, 46]. The same reasons prevail in low-income and high-income countries. But, as this study shows, the burden of economic constraints is more felt in low-income countries [47] and limited accessibility to medical care [13, 17]. Limited financial means are also a reason to purchase pharmaceutical drugs in IMPDs [14].

Affordability and the possibility to purchase quantities of drugs adapted to specific needs are additional reasons that may push people toward IMPDs [14, 48]. Besides affordability, people can be motivated by various factors, such as the availability of sellers irrespective of the time of the day, and the ability to purchase ‘prescription-only drugs’ without prescription [14, 49].

Another important point is that vendors often live in close proximity to the customers, sharing the same way of life [16, 49].

Based on Ministry of Health information, Benin has an average of 7.3 qualified health professionals (doctors, nurses and midwives) per 10,000 inhabitants. Cotonou is the city with the highest ratio, 29 qualified health professionals per 10,000 inhabitants [22], when the WHO recommends 23 qualified health professionals per 10,000 inhabitants as needed to provide the most essential maternal and child care [50]. Question about the lack of human resource does not arise in Cotonou. Factors identified in this study may differ from other city where there is a lack of health facilities. Several studies show that the distance to the health facility is a predictor of home treatment [51, 52], of access appropriate and effective health care [53] and also of childhood mortality [54]. It was found in this study that financial means was a factor associated with children home treatment. The financial means were also found in area where there is a lack of health facilities [31]. The conclusion is that distance from facility is not the main reason of the practice of home treatment in region that faces the lack of health facilities. Indeed, persons who live far from health facility have to spend money on transportation in addition to hospital expenses.

Authors reported that the continued existence of self-medication and then home treatment is favoured by the IMPD [11, 14] as people have access to the same drugs than in formal market, but at cheaper prices. Caregivers who purchased drugs in IMPD purchased more medicines than those who went to formal markets.

Information about the place of purchase was not asked in the case of consultation with a healthcare professional. Indeed, it would be interesting to study whether, after prescription, people still go to purchase drugs in IMPDs, as it was previously observed in Cotonou in a qualitative survey conducted from informal pharmaceutical drugs vendors [16].

### Limitations

Data were obtained from 340 children out of the 384 children expected. In this case, there is a loss of statistical power. Information collected was based on a past disease consequently the memory work was asked to the caregivers. Results must be considered while taking into account these elements. Nevertheless, sensitive analyses were done, and allowed to conclude there was no evidence for memory time-related bias.

## Conclusion

This study shows that malaria home treatment and use of informal drug market are at the centre of the healthcare practice of people in Cotonou, Benin. As patients who consult health-care professional also purchase prescribed drug in the IMPD, theses two phenomena must be considered separately.

On IMPD, a part of the medicines originate from the formal pharmaceutical sector in Benin, but the majority are coming informally from Ghana and Nigeria where they are legally distributed. Due to different drug distribution policies between countries, they are considered as false medicine in Benin. On February 24, 2017 as part of the 9th edition of Operation “PANGEA IX coordinated by Interpol, in partnership with the World Customs Organization (WCO), in the main popular market of Cotonou, 80 tons of medicines were seized and 109 people arrested. It is obvious that this action largely slowed down the informal market but, at the same time, reduced the capacity of the population to purchase medicine at low price. New studies are needed to better understand how people presently acquire pharmaceutical drugs, mainly for self-medication or home treatment.

## Additional files

**Additional file 1.** Sensitivity analysis based on chi-square test to highlight a possible memory bias related to time.

**Additional file 2.** Trade names of pharmaceutical drugs used by caregivers to treat children under twelve in Cotonou, Benin, May 2016.

## Abbreviations

ACT: artemisinin-based combination therapy
GPS: Global Positioning System
IMPD: informal market of pharmaceutical drugs
OR: odds ratio
WHO: World Health Organization
