# Supplementary material for: Home treatment and use of informal market of pharmaceutical drugs for the management of paediatric malaria in Cotonou, Benin
Source: Malar J. 2018 Oct 10;17:354. doi: 10.1186/s12936-018-2504-1 (PMC6180418; doi:10.1186/s12936-018-2504-1)
Supplement: Supplementary file 1 — Additional file 1. Sensitivity analysis based on chi-square test to highlight a possible memory bias related to time. [file 12936_2018_2504_MOESM1_ESM.docx]

Additional file 1 : Sensitivity analysis based on chi-square test to highlight a possible memory bias related to time.

| Date of the most recent episode of malaria in month | Forgetfulness of the name of the treatment used | Variation of the therapeutic class used |
| --- | --- | --- |
| <=1 versus >1 | p=0,706 | p=0,190 |
| <=2 versus >2 | p=0,418 | P=0,348 |
| <=3 versus >3 | p=0,742 | p=0,466 |
| <=4 versus >4 | p=0,342 | p=0,683 |
| <=5 versus >5 | p=0,185 | P=0,554 |
| <=6 versus >6 | P=0,393 | P=0,206 |
