# Supplementary material for: Home treatment and use of informal market of pharmaceutical drugs for the management of paediatric malaria in Cotonou, Benin
Source: Malar J. 2018 Oct 10;17:354. doi: 10.1186/s12936-018-2504-1 (PMC6180418; doi:10.1186/s12936-018-2504-1)
Supplement: Supplementary file 2 — Additional file 2. Trade names of pharmaceutical drugs used by caregivers to treat children under twelve in Cotonou, Benin, May 2016. [file 12936_2018_2504_MOESM2_ESM.docx]

Additional file 2: Trade names of pharmaceutical drugs used by caregivers to treat malaria in children under twelve years in Cotonou, Benin, May 2016 (N=256)

| Trade names cited | Healthcare Professional  n=71 | Home treatment | | Total |
| --- | --- | --- | --- | --- |
|  |  | Formal Market n=118 | IMPD  n=67 |  |
| PARACETAMOL | 14 | 49 | 51 | 114 |
| QUININE | 45 | 24 | 21 | 90 |
| EFFERALGAN | 10 | 14 | 2 | 26 |
| AMOXICILLINE | 6 | 9 | 9 | 24 |
| FER FOLDINE | 4 | 7 | 13 | 24 |
| CTA | 2 | 16 | 5 | 23 |
| LUFANTER | 4 | 8 | 2 | 14 |
| COFANTRINE | 3 | 7 | 0 | 10 |
| CHLOROQUINE | 0 | 2 | 5 | 7 |
| COARTEM | 2 | 3 | 1 | 6 |
| DOLIPRANE | 3 | 0 | 3 | 6 |
| ADVIL | 2 | 3 | 0 | 5 |
| ARTEFAN | 2 | 3 | 0 | 5 |
| BENDEX | 1 | 1 | 2 | 4 |
| MALOXINE | 1 | 2 | 1 | 4 |
| NIVAQUINE | 0 | 1 | 3 | 4 |
| NOVALGIN | 1 | 1 | 2 | 4 |
| PHILCO-MAX | 0 | 4 | 0 | 4 |
| CO-ARINATE | 2 | 1 | 0 | 3 |
| COMBIART | 0 | 1 | 2 | 3 |
| LUMARTEM | 0 | 2 | 1 | 3 |
| VERMOX | 0 | 2 | 1 | 3 |
| AMODIAQUINE | 0 | 2 | 0 | 2 |
| API PALU | 0 | 2 | 0 | 2 |
| ARTRIN | 2 | 0 | 0 | 2 |
| ASPEGIC | 1 | 1 | 0 | 2 |
| AUGMENTIN | 1 | 1 | 0 | 2 |
| BIMALARIL | 2 | 0 | 0 | 2 |
| BOSKA | 0 | 0 | 2 | 2 |
| CLAMOXYL | 2 | 0 | 0 | 2 |
| CLAMOXYL | 0 | 2 | 0 | 2 |
| CO-AMOX | 2 | 0 | 0 | 2 |
| TANZOL | 0 | 0 | 2 | 2 |
| ACLAV | 1 | 0 | 0 | 1 |
| AERIUS | 1 | 0 | 0 | 1 |
| AMPICILLINE | 0 | 1 | 0 | 1 |
| ANAFLAM | 0 | 0 | 1 | 1 |
| APDYL-H | 0 | 1 | 0 | 1 |
| ARTEMETHER | 1 | 0 | 0 | 1 |
| ARTEQUIN | 0 | 1 | 0 | 1 |
| ARTESUNATE | 1 | 0 | 0 | 1 |
| ASPIRINE | 0 | 1 | 0 | 1 |
| BACTOX | 1 | 0 | 0 | 1 |
| BACTRIM | 0 | 0 | 1 | 1 |
| BIODOXIN | 1 | 0 | 0 | 1 |
| BRUSTRAN-N | 0 | 1 | 0 | 1 |
| CAC 1000 | 1 | 0 | 0 | 1 |
| CETHER-L | 0 | 1 | 0 | 1 |
| CIPROFLOXACINE | 0 | 0 | 1 | 1 |
| CLAVUMOCID | 1 | 0 | 0 | 1 |
| COMBIMAL | 0 | 1 | 0 | 1 |
| DEPARASITANT | 1 | 0 | 0 | 1 |
| ERYTHROMYCINE | 1 | 0 | 0 | 1 |
| FERCEFOL | 0 | 1 | 0 | 1 |
| FERCEL | 0 | 0 | 1 | 1 |
| FLAGYL | 0 | 0 | 1 | 1 |
| FLUVERMAL | 1 | 0 | 0 | 1 |
| FOLIFER | 1 | 0 | 0 | 1 |
| FUMARATE FERREUX | 1 | 0 | 0 | 1 |
| GENVIT | 0 | 1 | 0 | 1 |
| HAEMOGLOBIN | 0 | 0 | 1 | 1 |
| HEMAFER | 1 | 0 | 0 | 1 |
| IBUMEX | 1 | 0 | 0 | 1 |
| IBUPROFENE | 0 | 1 | 0 | 1 |
| KINAL | 0 | 1 | 0 | 1 |
| LUMATE FORTE | 1 | 0 | 0 | 1 |
| MEBENDAZOLE | 0 | 1 | 0 | 1 |
| NURAVIT | 1 | 0 | 0 | 1 |
| OTRIVINE | 0 | 1 | 0 | 1 |
| PAIDOFEBRIL | 0 | 1 | 0 | 1 |
| PALUDRINE | 1 | 0 | 0 | 1 |
| PANOL | 0 | 1 | 0 | 1 |
| PARADOL | 0 | 1 | 0 | 1 |
| PASSION | 0 | 0 | 1 | 1 |
| PERVITAL | 1 | 0 | 0 | 1 |
| PTC | 0 | 0 | 1 | 1 |
| RANFERON | 1 | 0 | 0 | 1 |
| RHINALER | 1 | 0 | 0 | 1 |
| SMECTA | 1 | 0 | 0 | 1 |
| SUPER APPETIT | 1 | 0 | 0 | 1 |
| T-FER | 0 | 1 | 0 | 1 |
| TIMOFEROL | 1 | 0 | 0 | 1 |
| VITAFER | 1 | 0 | 0 | 1 |
